# Autoimmune uveitis: a retrospective analysis of 104 patients from a tertiary reference center

**DOI:** 10.1186/s12348-014-0017-9

**Published:** 2014-07-24

**Authors:** Marcella Prete, Silvana Guerriero, Rosanna Dammacco, Maria Celeste Fatone, Angelo Vacca, Francesco Dammacco, Vito Racanelli

**Affiliations:** 1 Department of Internal Medicine and Clinical Oncology, University of Bari Medical School, Piazza G. Cesare 11, Bari 70124, Italy; 2 Department of Basic Medical Sciences, Neuroscience and Sense Organs, University of Bari Medical School, Piazza G. Cesare 11, Bari 70124, Italy

**Keywords:** Autoimmune uveitis, Ankylosing spondyloarthritis, Behcet's disease, Class I and II HLA, Corticosteroids, Immunosuppressive drugs

## Abstract

**Background:**

The aim of this study was to identify the main features of a cohort of Caucasian patients with idiopathic (I) and systemic disease-associated (SDA) autoimmune uveitis (AU) who were followed up at a single tertiary reference center. The study consisted of a retrospective analysis of the demographic, clinical, and laboratory features and the response to treatment of 104 patients with AU evaluated between 2004 and 2013, with a median follow-up of 4.8 years. The primary outcome measure was the response to systemic treatment after 24 months of therapy. The data are expressed as the range, percentage, or mean ± standard error. Categorical variables were assessed by Fisher's exact test.

**Results:**

The mean age at diagnosis was 40.1 ± 17.8 years for men and 44.1 ± 15.3 years for women. There was a slight female predominance. Of the 104 patients, 72.1% had I-AU and 27.9% SDA-AU. The most frequent associations were with ankylosing spondyloarthritis, autoimmune thyroiditis, inflammatory bowel diseases, and Behcet's disease. Symptoms at presentation consisted of eye redness and pain (28.8%), decreased visual acuity (25.9%), and floaters (18.3%). Complications included cataracts (24%), retinal neovascularization (16.3%), chorio-retinal scars (10.6%), cystoid macular edema (8.6%), glaucoma/ocular hypertension (7.7%), epiretinal membranes (4.8%), and retinal detachment (3.8%). The prevalence of autoantibodies, mostly antinuclear antibodies, was comparable between the I-AU and SDA-AU groups. Fisher's exact test showed a direct correlation between patients with class I HLA B27, Cw8, B5 (51, 52), B51, or Cw2 and the presence of AU, whereas among patients with class II HLA, only DQ1 was a predisposing factor for AU. The therapeutic spectrum included corticosteroids and immunosuppressive agents, given either alone or in various combinations according to the severity of AU and the extent of the clinical response. Among the immunosuppressive drugs, azathioprine was preferentially used for anterior uveitis, and cyclosporine-A for intermediate and posterior uveitis. An assessment of the patients after 24 months of therapy showed a complete remission in 43.3% and a significant clinical improvement in 26.9%.

**Conclusions:**

At our tertiary reference center, the prevalence in Caucasian patients of I-AU was approximately 2.5-fold higher than that of SDA-AU. Our findings point to the need for a patient-tailored therapeutic approach according to the anatomic site and the severity of AU. Therapy should be prolonged, over a period of months and even up to 1–2 years, in order to achieve stable control of the disease and to prevent severe complications. The outcome of SDA-AU is also influenced by treatment of the underlying systemic disease. Additional controlled trials are needed to assess the efficacy and the long-term safety of both the prescribed therapeutic agents and their combinations.

## Background

Uveitis is an inflammatory process of the uvea, the vascular membrane of the eye that includes the iris, ciliary body, and choroid. Clinically, it can be classified into two groups: (a) *infectious*, e.g., bacterial endophthalmitis, toxoplasmosis, or herpetic retinopathy, in which there is an obvious infectious etiology, and (b) *non-infectious*, in which the pathophysiology is presumed to be autoimmune or immune-mediated in nature [[1], [2]]. In the latter form, a uveal component, whether tissue damage or a microbial trigger, stimulates the generation of antigen-specific T cells and/or autoantibodies that are believed to play a pathogenetic role [[3]], hence the term autoimmune uveitis (AU).

AU accounts for the majority of all cases of uveitis, is a major cause of severe visual impairment, and is responsible for 10%–15% of all cases of blindness in Western countries [[1]]. It can occur either alone (idiopathic autoimmune uveitis, I-AU) or as part of a systemic syndrome (systemic disease-associated autoimmune uveitis, SDA-AU) in which the eye is one of the several organs involved. In up to 50% of the cases, AU precedes or follows the onset of an autoimmune disease, such as one of the spondyloarthritides (including those complicating inflammatory bowel disorders and juvenile idiopathic arthritis), Behcet's disease, Vogt-Koyanagi-Harada (VKH) syndrome, systemic lupus erythematosus, sarcoidosis, autoimmune hepatitis, and multiple sclerosis [[4]]. The clinical heterogeneity of AU and the uncertainties regarding its pathogenesis make treatment challenging. For patients with SDA-AU, identification of the underlying systemic disease is advantageous when therapeutic decisions are to be made, whereas in patients with I-AU, a tailored approach may include corticosteroids, immunosuppressive agents, and biological drugs. However, in either form of AU, current therapeutic strategies are hampered by the paucity of randomized controlled trials and the absence of trials comparing the different therapeutic options [[5]].

In this paper, we summarize the main clinical, immunological, diagnostic, and therapeutic features of a relatively large group of Caucasian patients with AU who were followed up at a single tertiary reference center.

## Methods

### Patients

This study evaluated 104 patients with AU who were diagnosed between 2004 and 2013. Detailed records of their clinical history and ocular and systemic examinations, at presentation and throughout follow-up, were available for all of the patients.

Study patients underwent a complete ophthalmic assessment including best corrected visual acuity, intraocular pressure, indirect ophthalmoscopy, and slit-lamp biomicroscopy to examine the posterior segment and pars plana. Fluorescein angiography, indocyanine green angiography and/or optical coherence tomography, and/or ultrasound biomicroscopy were performed whenever complications of AU were suspected. Complete blood counts, erythrocyte sedimentation rate, C-reactive protein, liver and renal function tests, and serum protein electrophoresis were carried out as baseline investigations in all patients. Additional tests included typing for HLA class I and II phenotypes, serum C3 and C4 levels, anti-nuclear antibodies, anti-double-stranded DNA, rheumatoid factor and anti-cyclic citrullinated peptides, anti-thyroglobulin, anti-thyroperoxidase, and anti-neutrophil cytoplasmic antibodies with cytoplasmic- (c) or perinuclear (p)-staining. Bacterial, viral, fungal, and protozoal infections were excluded in all patients by targeted laboratory tests. Instrumental exams, such as chest X-ray, chest computed tomography, ultrasound examination of the upper and lower abdomen, radiographs of the skeleton, colonoscopy, and magnetic resonance imaging of the brain and the spine were performed whenever an association between AU and systemic diseases (such as multiple sclerosis or intracranial lymphoma) was suspected or if the exam was deemed clinically useful.

### Diagnosis

The diagnostic algorithm was as follows: first, uveitis was diagnosed, categorized, and graded according to the Standardization of Uveitis Nomenclature Working Group criteria [[6]]. Specifically, uveitis was classified in terms of anatomic localization, namely: (a) anterior (iritis, iridocyclitis, and anterior cyclitis); (b) intermediate (pars planitis, posterior cyclitis, and hyalitis); (c) posterior (focal, multifocal, or diffuse choroiditis, chorioretinitis, retinochoroiditis, retinitis, and neuroretinitis); (d) panuveitis (inflammation of the anterior chamber, vitreous, and retina or choroid). In addition, uveitis was categorized as acute, chronic, or recurrent according to its course, whether it was unilateral or bilateral, or granulomatous or non-granulomatous. The four aspects of intraocular inflammation (anterior chamber cells, anterior chamber flare, vitreous cells, and vitreous haze or debris) were ranked using an ordinal scale ranging from 0 to 4+ [[6]]. Second, patients were investigated for infectious etiologies, including tuberculosis, toxoplasmosis, syphilis, borreliosis, rickettsial infections, toxocariasis, herpes zoster virus, cytomegalovirus, Epstein-Barr virus, human immunodeficiency virus, and rubella. Patients who tested positive for any of these conditions were excluded.

Patients with anterior, intermediate, or posterior uveitis or panuveitis were considered to have I-AU if the following criteria were fulfilled: (i) all known causes of infectious uveitis had been ruled out, (ii) a systemic disease was found neither at the onset of uveitis nor during the median follow-up of 4.8 years.

### Therapy

The treatment algorithm differed according to the anatomic site and the severity of the AU. Obviously, patients with acute anterior AU who responded to topical therapy alone with disease quiescence did not require systemic treatment. For example, patients with idiopathic intermediate AU, good visual acuity, and lacking of complications were not treated as long as the disease remained stable. They did, however, undergo a periodic follow-up. Patients with recurrent or reactivated anterior AU, and especially those with I-AU, were treated with periocular subtenon injections (betamethasone phosphate, 3 mg/0.5 ml), either alone or in combination with oral corticosteroids (0.8–1 mg/kg/die). To achieve long-term control, particularly in patients with unilateral disease, triamcinolone (20 mg) was injected periocularly roughly every 6 months.

In the treatment of anterior SDA-AU or severe I-AU, one (rarely two) of the following immunosuppressive drugs was (were) administered in combination with corticosteroids (usually at the lower dose of 0.3–0.5 mg/kg/day): azathioprine (1 mg/kg/day), cyclosporine-A (2–3 mg/kg/day), cyclophosphamide (1 mg/kg/day), methotrexate (5–7.5 mg/weekly), and mycophenolate mofetil (1–1.5 g/day). The aim of these combinations was to achieve a steroid-sparing effect, and hence to minimize adverse events, but also to control the inflammation in case of corticosteroid failure. The combinations also implied a reasonable expectation of synergistic effects between the two or three drugs employed, justifying a lower than usual dose for each one. It should be emphasized that patients included in this survey were ‘difficult-to-treat’ cases. Indeed, they had been forwarded to our tertiary reference center because of poor responsiveness to conventional steroid therapies, and/or remarkable side effects, and/or frequent recurrences.

The same treatment combination of oral corticosteroids and immunosuppressive drugs was adopted in patients with intermediate AU and severe, bilateral involvement or complications such as cystoid macular edema or retinal vasculitis.

In patients with posterior uveitis or panuveitis, systemic immunomodulatory therapy was always required to achieve immediate and long-term disease control. When unilateral AU was not responsive to topical, periocular, or systemic therapy, intravitreal dexamethasone implants were performed. Finally, patients refractory to previous immunosuppressive drugs received biological therapies with antibodies to either anti-tumor necrosis factor-α or, in case of severe complications, anti-vascular endothelial growth factor.

Although all patients were followed-up at variable intervals, a final assessment in terms of therapeutic efficacy was carried out at 24 months, according to the following criteria: disease inactivity (grade 0), worsening activity (two-step increase in the level of inflammation or an increase from grade 3+ to 4+), improved activity (two-step decrease in the level of inflammation or a decrease to grade 0), and remission (inactive disease for ≥ 3 months after the discontinuation of all treatments for eye disease) [[7]].

### Statistical analyses

Statistical assessment was carried out using Prism (GraphPad Software), from the Statistical Package for Social Sciences (SPSS Inc., Chicago, IL, USA). The data are expressed as range, percentage, or mean ± standard error, as applicable.

The significance of the association between AU and HLA-class I and class II allotypes was calculated by Fisher's exact test, corrected for the alpha error, using a database on a control population of 212 HLA-I- and HLA-II-typed healthy unrelated blood donors. Both the *p* values and the odds ratio (OR) with the 95% confidence interval were calculated using the Statcalc program. Significance was defined as *p* < 0.05, with a relative risk >1.

## Results

**Figure 1:**
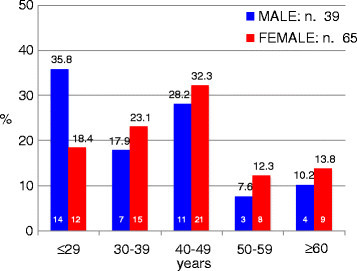
**Percentage distribution by age and gender of 104 patients with autoimmune uveitis (AU).** The number inside each bar indicates the number of patients corresponding to that age group and sex.

All patients were Caucasians, with a slight female predominance (F/M ratio, 1.7). The mean age at diagnosis was 40.1 ± 17.8 years (range 8–76) for men and 44.1 ± 15.3 years (range 14–73) for women. Among the 104 patients, 80 (76.9%) were younger than 50 years of age, including 26 patients (25%) who were younger than 30 years, 11 patients (10.6%) were between the ages of 50 and 59, and 13 patients (12.5%) were 60 years or older. Figure 1 summarizes the patient distribution according to gender and age group. Anterior uveitis was diagnosed in 48 patients (46.1%), posterior uveitis in 45 (43.2%), panuveitis in 6 (5.7%), and intermediate uveitis in 5 (4.8%).

**Figure 2:**
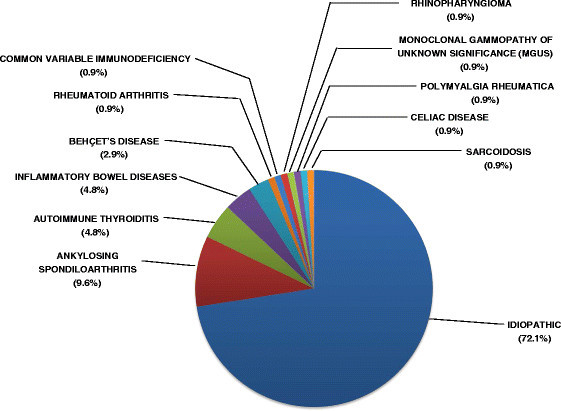
Clinical classification of 75 patients with idiopathic AU and 29 patients with systemic disease-associated AU.

I-AU was diagnosed in 75 patients (72.1%) and SDA-AU in the remaining 29 patients (27.9%). A systemic disease was already present at the onset of AU in 20 patients (19.2%) but was diagnosed during follow-up in the remaining 9 patients (8.6%). Associated diseases included ankylosing spondyloarthritis in ten patients (9.6%), autoimmune thyroiditis in five patients (4.8%), inflammatory bowel diseases in five patients (4.8%), and Behcet's disease in three patients (2.9%). Furthermore, there was one case (0.9%) of each of the following diseases: rheumatoid arthritis, common variable immunodeficiency, rhinopharyngioma, monoclonal gammopathy of unknown significance IgGK, polymyalgia rheumatica, celiac disease, and sarcoidosis (Figure 2).

**Figure 3:**
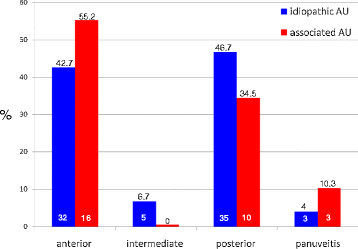
**Percentages of idiopathic and systemic disease-associated AU according to anatomic site in the cohort of 104 patients.** The number inside each bar indicates the number of patients belonging to the corresponding study group.

Among the 75 patients with I-AU, 32 (42.7%) had anterior uveitis, 5 (6.7%) had intermediate uveitis, 35 (46.7%) posterior uveitis, and the remaining 3 (4%) panuveitis. Of the 29 patients with SDA-AU, 16 (55.2%) had anterior uveitis, 10 (34.5%) had posterior uveitis, and 3 (10.3%) panuveitis. The anatomic distribution of AU is reported in Figure 3.

The spectrum of eye symptomatology at presentation ranged from the absence of symptoms to eye redness, ocular pain, light sensitivity, blurred vision, and photophobia and to declining visual acuity, scotoma, and floaters. The most common symptoms were eye redness and pain, which occurred in 38 patients (36.5%); a decrease of visual acuity was reported by 27 patients (25.9%); floaters were described by 19 patients (18.3%). In addition, while eye redness and pain and a decrease in visual acuity were the most common symptoms among patients with I-AU, eye redness and pain were most frequently determined in SDA-AU (Table 1). Symptoms occurred suddenly and worsened rapidly in 37 patients (35.6%) but developed gradually and assumed a chronic and recurrent course in 67 patients (64.4%). They were detected in one eye in 57 patients (54.8%) and in both eyes in the remaining 47 (45.2%).

**Table 1:** Symptoms at presentation in 104 patients with autoimmune uveitis

**Symptoms**	**Idiopathic autoimmune uveitis(*n*** **= 75 pts)**	**Systemic disease-associated autoimmune uveitis(*n*** **= 29 pts)**	**Overall autoimmune uveitis(*n*** **= 104 pts)**
Eye redness/eye pain	23 (30.6%)	15 (51.7%)	38 (36.5%)
Decrease in visual acuity	23 (30.6%)	4 (13.8%)	27 (25.9%)
Floaters	15 (20.0%)	4 (13.8%)	19 (18.3%)
Blurred vision	8 (10.7%)	1 (3.4%)	9 (8.6%)
Photophobia	3 (4.0%)	3 (10.3%)	6 (5.8%)
Scotoma	4 (5.3%)	1 (3.4%)	5 (4.8%)

The complications that occurred during follow-up consisted of cataracts in 25 patients (24%), retinal neovascularization in 17 patients (16.3%), chorio-retinal scars in 11 patients (10.6%), cystoid macular edema in 9 patients (8.6%), and glaucoma in 8 patients (7.7%). The prevalence of each complication was roughly comparable between patients with I-AU and those with SDA-AU (Table 2). By contrast, retinal neovascularization, epiretinal membranes, and retinal detachment were detected only in patients with posterior uveitis or panuveitis. Cystoid macular edema was most frequently present in intermediate forms of AU (Table 2).

**Table 2:** Complications determined in 104 patients with autoimmune uveitis

**Complications**	**Idiopathic autoimmune uveitis(75 pts)**	**Systemic disease-associated autoimmune uveitis(29 pts)**	**Anterior autoimmune uveitis(48 pts)**	**Intermediate autoimmune uveitis(5 pts)**	**Posterior autoimmune uveitis(45 pts)**	**Panuveitis(6 pts)**	**Overall autoimmune uveitis(104 pts)**
Cataract	18 (24.0%)	7 (24.1%)	12 (25.0%)	1 (20.0%)	10 (22.2%)	2 (33.3%)	25 (24.0%)
Retinal neovascularization	13 (17.3%)	4 (13.8%)	0 (0.0%)	0 (0.0%)	13 (28.9%)	4 (66.6%)	17 (16.3%)
Chorio-retinal scars	7 (9.3%)	4 (13.8%)	3 (6.2%)	0 (0.0%)	7 (15.5%)	1 (16.6%)	11 (10.6%)
Cystoid macular edema	7 (9.3%)	2 (6.9%)	0 (0.0%)	4 (80.0%)	4 (8.9%)	1 (16.6%)	9 (8.6%)
Glaucoma/ocular hypertension	6 (8.0%)	2 (6.9%)	3 (6.2%)	0 (0.0%)	4 (8.9%)	1 (16.6%)	8 (7.7%)
Epiretinal membranes	4 (5.3%)	1 (3.4%)	0 (0.0%)	0 (0.0%)	3 (6.7%)	2 (33.3%)	5 (4.8%)
Retinal detachment	2 (2.7%)	2 (6.9%)	0 (0.0%)	0 (0.0%)	2 (4.4%)	2 (33.3%)	4 (3.8%)

Laboratory examination showed an increased erythrocyte sedimentation rate (>10 mm/h) and increased serum C-reactive protein (>3 mg/L) in 30 patients (28.8%). In this group, 22 patients (73.3%) had SDA-AU and 8 (26.6%) I-AU. Twenty-seven patients (25.9%) tested positive for anti-nuclear antibodies, one (0.9%) tested positive for anti-double stranded DNA, three (2.9%) for anti-cyclic citrullinated peptides, and two (1.9%) for (p) anti-neutrophil cytoplasmic antibodies. These autoantibodies were present in 17 patients (22.7%) with I-AU and in 8 patients (27.6%) with SDA-AU. There was no significant correlation between the above laboratory parameters and the clinical subtype of AU, except that increases in erythrocyte sedimentation rate and C-reactive protein were detected more frequently in patients with SDA-AU.

Characterization of class I HLA showed that B27 (OR, 6.6; *p* = 0.001), Cw8 (OR, 6.1; *p* = 0.027), B5(51,52) (OR, 4.4; *p* < 0.0001), B(51) (OR, 3.7; *p* < 0.0008), and Cw2 (OR, 3.2; *p* = 0.019) were associated with the presence of AU. For class II HLA, however, only DQ1 was a predisposing factor for AU (OR 3.3; *p* < 0.0003) (Table 3). None of the patients positive for DQ1 had diabetes or celiac disease.

**Table 3:** Association between autoimmune uveitis and class I and II HLA antigens

	**OR**	**Fischer (*p*** **value)**
**HLA-I**		
A1	0.31	0.0025
A28 (68)	12.4	0.01
B27	6.6	0.001
B5 (51,52)	4.4	<0.0001
B (51)	3.7	0.0008
Cw2	3.2	0.019
Cw7	0.4	0.02
Cw8	6.1	0.027
**HLA-II**		
DR3	0.05	0.0025
DR7	0.46	0.043
DQ1	3.3	0.0003
DQ2	0.5	0.049
DR5	0.26	0.01

Treatment regimens at diagnosis included the use of corticosteroids for all patients (Table 4). Five patients (4.8%) were given periocular subtenon injections and/or systemic corticosteroids alone (prednisone: 1 mg/kg/daily) as an induction treatment, which was then tapered in step with a favorable clinical response. Oral corticosteroids, in combination with one or two immunosuppressive drugs, were administered to 99 patients (95.1%). Of these, 81 patients (77.9%) were given a single immunosuppressive agent (azathioprine or cyclosporine-A or cyclophosphamide or methotrexate), either because of refractoriness to corticosteroids or with a steroid-sparing aim. These patients had bilateral (without severe complications) or monolateral AU.

**Table 4:** Systemic treatment at baseline according to anatomic and clinical classifications of autoimmune uveitis (AU)

**Treatment at baseline**	**Total number(104 pts)**	**Anterior autoimmune uveitis(48 pts)**	**Intermediate autoimmune uveitis(5 pts)**	**Posterior autoimmune uveitis(45 pts)**	**Panuveitis(6 pts)**	**Idiopathic autoimmune uveitis(75 pts)**	**Systemic disease-associated autoimmune uveitis(29 pts)**
Corticosteroids	5 (4.8%)	4 (80%)	1 (20%)	0 (0%)	0 (0%)	4 (80%)	1 (20%)
Corticosteroids + Azathioprine	36 (34.6%)	31 (86.1%)	1 (2.7%)	4 (11.1%)	0 (0.0%)	24 (66.6%)	12 (33.3%)
Corticosteroids + Cyclosporine-A	35 (33.6%)	9 (25.7%)	2 (5.7%)	24 (68.5%)	0 (0%)	29 (82.8%)	6 (17.1%)
Corticosteroids + Cyclophosphamide	8 (7.6%)	0 (0%)	0 (0%)	7 (87.5%)	1 (12.5%)	6 (75%)	2 (25%)
Corticosteroids + Methotrexate	2 (1.9%)	1 (50%)	0 (0%)	1 (50%)	0 (0%)	0 (0%)	2 (100%)
Corticosteroids + Combined immunosuppressive therapy	18 (17.3%)	3 (16.6%)	1 (5.5%)	9 (50%)^a^	5 (27.7%)	12 (66.6%)	6 (33.3%)

^a^In three patients, corticosteroids were given as an FDA-approved dexamethasone intravitreal implant.

Thirty-six patients (34.6%) were treated with azathioprine: 24 of them (66.6%) had I-AU and the remaining 12 (33.3%) SDA-AU. Anterior AU was diagnosed in the large majority of these patients (31, 86.1%). Cyclosporine-A was given to 35 patients (33.6%): 29 patients (82.8%) with I-AU and 6 (17.1%) with SDA-AU. In the majority of cases (24 patients, 68.5%), posterior uveitis was diagnosed. Only eight patients (7.6%) were given cyclophosphamide: six of them (75%) had I-AU and the remaining two (25%) SDA-AU, refractory to other immunosuppressive drugs. Methotrexate was administered to two patients (1.9%), both with AU associated with rheumatoid arthritis. Mycophenolate mofetil was never used as a single agent.

A combined therapeutic approach with any two immunosuppressants, including azathioprine, cyclosporine-A, cyclophosphamide, mycophenolate mofetil, and methotrexate was adopted in 18 patients (17.3%) with severe complications at diagnosis or with persistently active or recurrent disease. In the three patients with anterior AU, corticosteroids were given as a Federal Drug Administration-approved intravitreal biodegradable implant (Ozurdex®) that slowly released dexamethasone (Table 4). Finally, monoclonal antibodies with either anti-tumor necrosis factor-α or anti-vascular endothelial growth factor activity were administered to ten patients (9.6%) with AU refractory to previous immunosuppressive drugs and to three patients (2.9%) with retinal neovascularization and cystoid macular edema.

According to the definitions described in the ‘Methods’ section, an assessment of the patients after 24 months of therapy showed that 45 of them (43.3%) achieved a complete remission and 28 (26.9%) a significant improvement, while in 31 (29.8%), the disease worsened. In the 36 patients (34.6%) with I-AU who achieved remission, treatment was gradually tapered and then discontinued in the following 12–24 months. In the nine patients (8.6%) with SDA-AU who achieved disease remission, corticosteroids were tapered and discontinued in the following 12 months, but immunosuppressive drugs were maintained to a lower dosage. In the 18 patients (17.3%) with I-AU that showed improvement, the administration of corticosteroids was prolonged for several months, then tapered, and finally discontinued in the following 12 months; immunosuppressive drugs were, however, maintained until remission. The same treatment modality was employed in ten patients (9.6%) with SDA-AU, who likewise improved when the immunosuppressive drugs were maintained. Finally, in the 31 patients with worsening disease (29.8%), including 21 with I-AU and 10 with SDA-AU, the therapeutic approach was switched to one based on monoclonal antibodies.

## Discussion

In agreement with a number of previously published reports [[1], [2], [8]–[11]], our study confirmed that AU frequently affects young adults, with a slight prevalence in females. In a large series encompassing over 2,600 patients, anterior uveitis (including infectious uveitis) was the most common entity, occurring in 59.9% [[4]]. This finding was confirmed in our case series, in which 48 patients had anterior uveitis (46.1%); specifically, anterior forms accounted for the majority (55.2%) of the SDA-AU cases, whereas posterior forms were prevalent in I-AU (46.7%). Obviously, the percentages of idiopathic forms in the various studies are influenced by the variable prevalence of infectious and non-infectious entities in different parts of the world as well as by the environmental, racial, and socioeconomic factors affecting the populations studied.

The anatomic classification of AU together with the standardized set of criteria for grading intraocular inflammation proposed by the Standardization of Uveitis Nomenclature Working Group is suitable for reporting clinical data and assessing the effectiveness of therapy [[6]]. As such, it is the main reference used by internists and ophthalmologists. Nevertheless, it has several limitations and uncertainties regarding forms of AU such as pars planitis, neuroretinitis, and anterior-intermediate uveitis, as recently emphasized [[12]].

A clinical classification, on the other hand, by separating I-AU from SDA-AU, provides important information that can be useful in terms of prognostic implications and therapeutic choice. Given that in our cohort, 72.1% of the patients had I-AU in the absence of other autoimmune manifestations, it is unclear whether I-AU is truly an organ-specific autoimmune disease. In spite of the relatively frequent association of AU with systemic diseases, no general consensus is available regarding the initial diagnostic work-up of patients with AU in the absence of a known systemic disease.

An important point stemming from our series analysis is the limited usefulness, if any, of the search for immunological serum markers in the identification of those patients with I-AU who are at risk for the development of an autoimmune-type systemic disease. None of the patients who tested positive for anti-nuclear antibodies met the diagnostic criteria for any of the rheumatic diseases, and in the following 24 months, they were in fact considered to have I-AU. However, the role of serological markers such as rheumatoid factor, anti-cyclic citrullinated peptides, anti-nuclear antibodies, and anti-neutrophil cytoplasmic antibodies in determining the risk of developing a systemic autoimmune disease has not been extensively studied in patients with I-AU [[13]]. To date, only one study has demonstrated the clinical usefulness of anti-neutrophil cytoplasmic antibodies and rheumatoid factor screening in identifying patients with idiopathic scleritis who are at risk of developing systemic diseases such as rheumatoid arthritis and Wegener's granulomatosis [[14]]. Thus, in patients with I-AU, laboratory evaluation, including serological tests for autoimmune diseases, has a limited role (if any) in diagnosis and follow-up.

A potentially useful prognostic factor may instead be the typing of HLA class I and II antigens, given the strong association of clearly defined AU with some allotypes. Strong HLA class I and class II associations have been reported for some types of uveitis. Striking examples are the associations of sympathetic ophthalmia and VKH syndrome with HLA-DR4, of VKH with HLA-DQ4, and of birdshot retinochoroidopathy with HLA-A29. In these conditions, the relative risk ranges from 49 to 224, depending on the study. HLA B27 was first recognized as a risk factor for acute anterior uveitis associated with ankylosing spondyloarthropathies [[15]]. Pars planitis was shown to be associated with an increased frequency of HLA-DR2 (suballele, −DR15, HLA-DR51) and HLA-DR17 [[14], [16]]. An association between DRB1and idiopathic intermediate uveitis has also been reported [[17]]. HLA associations provide support for the role of an autoimmune mechanism in AU, in that HLA molecules select and present antigens for recognition by T cells. Thus, an autoimmune T cell response is triggered only if a self-antigen is recognized in the context of a restricted HLA molecule.

Nonetheless, our results are at partial variance from the abovementioned studies. As reported herein, we found an association between AU and the class I HLA antigens B27, Cw8, B5(51,52), B5(51), and Cw2. Among the class II HLA antigens, only DRQ1 was shown to be a predisposing factor for AU. A likely explanation for the discrepancies with the literature data is the prevalence in our series of anterior and posterior uveitis (rather than panuveitis and intermediate uveitis), which occurred in roughly similar numbers of patients. An additional feature of our case series is the large number of patients with I-AU; the ratio of these patients to those with SDA-AU was nearly 3:1. Clearly, an appropriate statistical evaluation of the possible association of HLA type with each of the uveitis patterns included in this study would have provided important information. However, the relatively low number of patients affected with certain uveitis entities hindered this type of analysis; for example, there were only three patients with AU and Behcet's disease. Therefore, we instead decided to consider all SDA-AU patients as a single group, a decision justified by the fact that they shared the diagnosis of AU.

The pathogenesis of AU is still largely undefined but is likely polyfactorial. An aberrant T cell-mediated immune response, with breakdown of the blood-retinal barrier, has been hypothesized. This mechanism implies direct immunological action against retinal or cross-reactive antigens, triggered by infections and/or inflammation [[18]]. The retinal antigens involved are most likely melanocyte components [[19]] or tyrosinase or tyrosinase-related proteins [[20]]. CD4+ T cells are thought to play a pivotal role in the development and maintenance of AU [[18]]. Experimental data in mice indicate that the deletion of signal transducers and activators of transcription-3 and of retinoic acid receptor-related orphan nuclear receptors RORγt and RORα in CD4+ T cells can prevent the onset of experimental posterior AU [[21]]. It has also been shown in AU that Th1 and Th2 cells exhibit a dual function, both pathogenetic and protective, whereas Th9 and Th17 play only a pathogenetic role [[18]].

Recent evidence of the involvement of the innate immune response and the absence of specific autoantibodies suggests that AU is an autoinflammatory disease [[22]]. It has indeed been demonstrated that Toll-like receptors 2 and 4, expressed on antigen-presenting cells of the iris, choroid, and ciliary body (anterior AU) [[23]], can be activated by bacterial components (i.e., the peptidoglycans of gram-positive bacteria and the lipopolysaccharides of gram-negative bacteria) and provide the link between innate and cell-mediated immune responses [[24]]. Following their activation and polyclonal expansion, Th1 and Th17 cells escape tolerance mechanisms, probably because of the reduced number and impaired function of T regulatory cells [[24]]. This mechanism seems to be involved in Behcet's disease [[25]] and VKH syndrome [[26]], since in these patients, an increase in the T regulatory cell population and restoration of its functional state have been observed following therapy [[27]].

Based on the hypothesized pathogenetic mechanism, the use of immunosuppressive drugs seems justified. In fact, corticosteroids and cyclophosphamide block NFκB (nuclear factor kappa light chain enhancer of activated B cells signaling pathway); cyclosporine-A and mycophenolate mofetil act on CD4+ T cells, and specifically on signal transducers and activators of transcription-3 (STAT-3) and nuclear factor of activated T cells (NF-AT), which are mostly involved in posterior AU; AZA neutralizes CD8+ T cells and natural killer cells; monoclonal antibodies target specific cytokines, such as tumor necrosis factor-α and interleukins 1 and 6. Future therapeutic approaches to AU will probably be directed at Toll-like receptor pathways [[24]] and other specific cytokines. In the meantime, there are as yet no specific recommendations regarding the drugs of choice, their optimal combination, and the length of their administration for each type of AU [[28]]. However, there is clearly a need for more suitable combinations of the drugs currently in use - with improved efficacy and fewer, if any, side effects - thus inducing more durable remissions than presently obtained with long-term corticosteroid and immunosuppressive therapies [[29]].

The Standardization of Uveitis Nomenclature Working Group Guidelines recommend the use of corticosteroids as first-line therapy for patients with active uveitis [[6]]. However, it is well known that the long-term administration of corticosteroids is associated with numerous adverse events, including cataract, glaucoma, and metabolic disorders. Alternatively, immunosuppressive drugs (cyclosporine-A, rapamycin), cytotoxic agents (cyclophosphamide), and antimetabolites (azathioprine, methotrexate) are given as steroid-sparing agents [[7], [29]].

Our experience indicates that (i) the best therapeutic approach depends on the severity of the disease; (ii) for patients with I-AU, the choice of a specific immunosuppressive drug is based on the anatomic classification and grading of intraocular inflammation, while for SDA-AU the preferred immunosuppressive drug(s) are the same as those administered for the underlying systemic disease, (iii) immunosuppressive therapy should be carefully modulated during follow-up to avoid troublesome complications, (iv) maintenance immunosuppressive therapy should be prolonged for a period of months, and sometimes for up to on1–2 years, in order to achieve stable control of the disease.

Accordingly, we suggest that, while patients with unilateral AU should initially be given periocular corticosteroid injections, receiving systemic treatment in case of poor response, systemic corticosteroids should be administered from the very beginning in case of persistent disease activity, worsening in the same eye, or extension to both eyes and/or complications at treatment onset. Immunosuppressive therapy, on the other hand, should be started either as a steroid-sparing procedure or when corticosteroids fail to control the inflammation (persistent or recurrent disease, extension in the same eye or bilaterally), and obviously when AU is associated with an underlying autoimmune systemic disease. In case of frequent recurrences and/or the development of complications, our preferred approach is combined immunosuppressive therapy. Monoclonal antibodies are an option for patients with sight-threatening complications, patients refractory to other immunosuppressive drugs, and/or to control severe systemic autoimmune diseases.

Among the immunosuppressive drugs, azathioprine is usually our first choice for the treatment of anterior uveitis, and cyclosporine-A for intermediate and posterior uveitis. Cyclophosphamide should be avoided in women of childbearing age, due to its potential effects on fertility. As with corticosteroids, we try to modulate immunosuppressive drugs according to the degree of intraocular inflammation and the possible occurrence of complications and/or recurrences and/or disease extension in the same eye or bilaterally. In case of improvement/remission, corticosteroids are gradually tapered until their withdrawal, with a subsequent reduction in the use of immunosuppressive drugs.

Conversely, a combined immunosuppressive therapy is our choice in case of worsening, in order to spare corticosteroid administration and to achieve better disease control, thus preventing the development of serious complications. If remission is obtained, we prolong immunosuppressive treatment (without corticosteroids) for several months to reduce the probability of recurrence. As reported under the ‘Results’ section and summarized in Tables 4 and 5, the adoption of these therapeutic measures closely reflecting those described in recent reports [[7], [28]] allowed us to yield a good response (remission + improvement) in 73 patients (70.2%) at a mean follow-up of 24 months.

**Table 5:** Response to treatment according to anatomic classification of autoimmune uveitis

**Treatment**	**Remission**				**Improvement**				**Worsening**
**(*n*, %)**	**(45 pts, 43.3%)**				**(28 pts, 26.9%)**				**(31 pts, 29.8%)**
	**Anterior**	**Intermediate**	**Posterior**	**Panuveitis**	**Anterior**	**Intermediate**	**Posterior**	**Panuveitis**	
Corticosteroids (5, 4.8)	2		1		1				1
Corticosteroids + Azathioprine (36, 34.6)	9	1			7	1	2	1	15
Corticosteroids + Cyclosporin-A (35, 34.6)	1	1	8		2	1	8	1	12
Corticosteroids + Cyclophosphamide (8, 7.6)			3	1			1	1	2
Corticosteroids + Methotrexate (2, 1.9)	1		1						1
Corticosteroids + combined immuno-suppressive therapy (8, 7.6)	1	1	2	2			1	1	
Anti-tumor necrosis factor-α (7, 6.7)	1		3	3					0
Anti-vascular endothe-lial growth factor (3, 2.9)			2	1					0

Finally, we would like to emphasize two important points of our series analysis. The first one is the association found between AU and the class I HLA antigens B27, Cw8, B5(51,52), B5(51), and Cw2. Among the class II HLA antigens, only DRQ1 was shown to be a predisposing factor for AU. The second point is the limited usefulness, if any, of the search for immunological serum markers in the identification of those patients with I-AU who are at risk for the development of an autoimmune-type systemic disease.

## Conclusions

There are a number of issues that remain to be addressed. First of all, the majority of the studies dealing with AU, often conducted on a relatively small number of patients, are retrospective and only a few of them have comparatively established the effectiveness of the different therapeutic regimens. Thus, additional controlled trials are needed to assess the efficacy and long-term safety of the agents considered herein. Second, a standard definition of success should be established when biological agents are employed. Third, the classification of AU is still nebulous and vague, such that most studies include a heterogeneous array of patients with more than one specific diagnosis, which in turn complicates any therapeutic assessment. Additional reliable information is also required concerning the risks and benefits of systemic treatments in children and in women of childbearing age, as well as in patients with no associated systemic disease.

## Abbreviations

AU: autoimmune uveitis

I-AU: idiopathic autoimmune uveitis

SDA-AU: systemic disease-associated autoimmune uveitis

VKH: Vogt-Koyanagi-Harada syndrome

## Competing interests

The authors declare that they have no competing interests.

## Authors' contributions

MP, VR, and FD conceived and designed the study and wrote the manuscript. SG, RD, and MCF participated in the data collection, ophthalmological evaluation, and follow-up of the patients. AV conceived the study and critically revised the manuscript. All authors read and approved the final manuscript.

## References

[B1] Gritz DC Wong IG Incidence and prevalence of uveitis in Northern California; the Northern California Epidemiology of Uveitis Study Ophthalmology 2004 4 491 500 10.1016/j.ophtha.2003.06.014 15019324

[B2] Kitamei H Kitaichi N Namba K Kotake S Goda C Kitamura M Miyazaki A Ohno S Clinical features of intraocular inflammation in Hokkaido, Japan Acta Ophthalmol 2009 4 424 428 10.1111/j.1755-3768.2008.01282.x 18652578

[B3] Caspi RR A look at autoimmunity and inflammation in the eye J Clin Invest 2010 4 3073 3083 10.1172/JCI42440 20811163 PMC2929721

[B4] Barisani-Asenbauer T Maca SM Mejdoubi L Emminger W Machold K Auer H Uveitis - a rare disease often associated with systemic diseases and infections - a systematic review of 2619 patients Orphanet J Rare Dis 2012 4 57 10.1186/1750-1172-7-57 22932001 PMC3503654

[B5] Rathinam SR Namperumalsamy P Global variation and pattern changes in epidemiology of uveitis Indian J Ophthalmol 2007 4 173 83 10.4103/0301-4738.31936 17456933

[B6] Jabs DA Nussenblatt RB Rosenbaum JT Standardization of uveitis nomenclature for reporting clinical data. Results of the First International Workshop Am J Ophthalmol 2005 4 509 516 10.1016/j.ajo.2005.03.057 16196117 PMC8935739

[B7] Larson T Nussenblatt RB Sen HN Emerging drugs for uveitis Expert Opin Emerg Drugs 2011 4 309 322 10.1517/14728214.2011.537824 21210752 PMC3102121

[B8] Mercanti A Parolini B Bonora A Lequaglie Q Tomazzoli L Epidemiology of endogenous uveitis in north-eastern Italy. Analysis of 655 new cases Acta Ophthalmol Scand 2001 4 64 8 10.1034/j.1600-0420.2001.079001064.x 11167291

[B9] Keino H Nakashima C Watanabe T Taki W Hayakawa R Sugitani A Okada AA Frequency and clinical features of intraocular inflammation in Tokyo Clin Experiment Ophthalmol 2009 4 595 601 10.1111/j.1442-9071.2009.02102.x 19702710

[B10] Jones NP (2013): The Manchester Uveitis clinic: the first 3000 patients-epidemiology and casemix. Ocul Immunol Inflamm Dec 2 Epub ahead of print) 10.3109/09273948.2013.855799 24295124

[B11] Pan J Kapur M McCallum R Noninfectious immune-mediated uveitis and ocular inflammation Curr Allergy Asthma Rep 2014 4 409 10.1007/s11882-013-0409-1 24338488

[B12] Khairallah M Are the Standardization of the Uveitis Nomenclature (SUN) Working Group criteria for codifying the site of inflammation appropriate for all uveitis problems? Limitations of the SUN Working Group classification Ocul Immunol Inflamm 2010 4 2 4 10.3109/09273940903348835 20128640

[B13] Banares A Jover JA Benitez F-G del Castillo JM Garcia J Vargas E Hernandez-Garcia C Patterns of uveitis as a guide in making rheumatologic and immunologic diagnoses Arthritis Rheum 1997 4 358 370 10.1002/art.1780400221 9041948

[B14] Lin P Bhullar SS Tessler HH Goldstein DA Immunologic markers as potential predictors of systemic autoimmune disease in patients with idiopathic scleritis Am J Ophthalmol 2008 4 463 471 10.1016/j.ajo.2007.09.024 18061135

[B15] Martin TM Rosenbaum JT An update on the genetics of HLA B27-associated acute anterior uveitis Ocul Immunol Inflamm 2011 4 108 114 10.3109/09273948.2011.559302 21428748 PMC3083239

[B16] Oruc S Duffy BF Mohanakumar T Kaplan HJ The association of HLA class II with pars planitis Am J Ophthalmol 2001 4 657 659 10.1016/S0002-9394(00)00863-1 11336946

[B17] Atan D Heissigerova J Kuffova L Hogan A Kilmartin DJ Forrester JV Bidwell JL Dick AD Churchill AJ Tumor necrosis factor polymorphisms associated with tumor necrosis factor production influence the risk of idiopathic intermediate uveitis Mol Vis 2013 4 184 195 23378732 PMC3559088

[B18] Horai R Caspi RR Cytokines in autoimmune uveitis J Interferon Cytokine Res 2011 4 733 744 10.1089/jir.2011.0042 21787221 PMC3189550

[B19] Sugita S Takase H Taguchi C Imai Y Kamoi K Kawaguchi T Sugamoto Y Futagami Y Itoh K Mochizuki M Ocular infiltrating CD4+ T cells from patients with Vogt-Koyanagi-Harada disease recognize human melanocyte antigens Invest Ophthalmol Vis Sci 2006 4 2547 2554 10.1167/iovs.05-1547 16723469

[B20] Damico FM Cunha-Neto E Goldberg AC Iwai LK Marin ML Hammer J Kalil J Yamamoto JH T-cell recognition and cytokine profile induced by melanocyte epitopes in patients with HLA-DRB1*0405-positive and -negative Vogt-Koyanagi-Harada uveitis Invest Ophthalmol Vis Sci 2005 4 2465 2471 10.1167/iovs.04-1273 15980237

[B21] Yu CR Lee YS Mahdi RM Surendran N Egwuagu CE Therapeutic targeting of STAT3 (signal transducers and activators of transcription 3) pathway inhibits experimental autoimmune uveitis PLoS One 2012 4 e29742 10.1371/journal.pone.0029742 22238646 PMC3252323

[B22] Lee RW Dick AD Current concepts and future directions in the pathogenesis and treatment of non-infectious intraocular inflammation Eye (Lond) 2012 4 17 28 10.1038/eye.2011.255 21960067 PMC3259588

[B23] Chang JH McCluskey PJ Wakefield D Toll-like receptors in ocular immunity and the immunopathogenesis of inflammatory eye disease Br J Ophthalmol 2006 4 103 108 10.1136/bjo.2005.072686 16361678 PMC1856909

[B24] Pratap DS Lim LL Wang JJ Mackey DA Kearns LS Stawell RJ Burdon KP Mitchell P Craig JE Hall AJ Hewitt AW The role of toll-like receptor variants in acute anterior uveitis Mol Vis 2011 4 2970 2977 22128242 PMC3224833

[B25] Nanke Y Kotake S Goto M Ujihara H Matsubara M Kamatani N Decreased percentages of regulatory T cells in peripheral blood of patients with Behcet's disease before ocular attack: a possible predictive marker of ocular attack Mod Rheumatol 2008 4 354 358 10.3109/s10165-008-0064-x 18427720

[B26] Chen L Yang P Zhou H He H Ren X Chi W Wang L Kijlstra A Diminished frequency and function of CD4 + CD25high regulatory T cells associated with active uveitis in Vogt-Koyanagi-Harada syndrome Invest Ophthalmol Vis Sci 2008 4 3475 3482 10.1167/iovs.08-1793 18421089

[B27] Ruggieri S Frassanito MA Dammacco R Guerriero S Treg lymphocytes in autoimmune uveitis Ocul Immunol Inflamm 2012 4 255 261 10.3109/09273948.2012.681830 22564107

[B28] Pato E Munoz-Fernandez S Francisco F Abad MA Maese J Ortiz A Carmona L Systematic review on the effectiveness of immunosuppressants and biological therapies in the treatment of autoimmune posterior uveitis Semin Arthritis Rheum 2011 4 314 323 10.1016/j.semarthrit.2010.05.008 20656330

[B29] Levy RA de Andrade FA Foeldvari I Cutting-edge issues in autoimmune uveitis Clin Rev Allergy Immunol 2011 4 214 223 10.1007/s12016-011-8267-x 21913066

